# Identification of Five Ferroptosis-Related LncRNAs as Novel Prognosis and Diagnosis Signatures for Renal Cancer

**DOI:** 10.3389/fmolb.2021.763697

**Published:** 2022-01-18

**Authors:** Xiangjun Shu, Zaiqi Zhang, Zhi-Yong Yao, Xiao-Liang Xing

**Affiliations:** ^1^ School of Public Health and Laboratory Medicine, Hunan University of Medicine, Huaihua, China; ^2^ The First Affiliated Hospital, Hunan University of Medicine, Huaihua, China

**Keywords:** renal cancer, ferroptosis, differentially expressed lncRNAs, prognosis, diagnosis

## Abstract

**Background:** Ferroptosis is a novel regulated cell death that is characterized by iron-dependent oxidative damage. Renal cancer is the second most common cancer of the urinary system, which is highly correlated with iron metabolism. The aim of our present study was to identify suitable ferroptosis-related prognosis signatures for renal cancer.

**Methods:** We downloaded the RNA-seq count data of renal cancer from The Cancer Genome Atlas database and used the DESeq2, Survival, and Cox regression analyses to screen the prognosis signatures.

**Results:** We identified 5 ferroptosis-related differentially expressed lncRNAs (FR-DELs) (DOCK8-AS1, SNHG17, RUSC1-AS1, LINC02609, and LUCAT1) to be independently correlated with the overall survival (OS) of patients with renal cancer. The risk assessment model and diagnosis model constructed by those 5 FR-DELs could well predict the outcome and the diagnosis of renal cancer.

**Conclusion:** Our present study not only suggested those 5 FR-DELs could be used as prognosis and diagnosis signatures for renal cancer but also provided strategies for other cancers in the screening of ferroptosis-related biomarkers.

## Introduction

Renal cell cancer (kidney cancer) is the second most common cancer of the urinary system. In 2020, there were almost 430,000 new cases (accounting for 2.2% of all new cases) and 180,000 deaths due to renal cancer (accounting for 1.8% of all death cases) (Sung et al., 2021). Renal cell carcinoma (RCC) is a heterogeneous group of cancers originating from the renal epithelium, which comprises the major subtype clear cell RCC (KIRC) and papillary RCC (KIRP). The most common pathologic type is KIRC, which accounts for about 70%–80% of renal cell cancer (Lipworth et al., 2016; Low et al., 2016). A previous study indicated that approximately 40% of patients with advanced KIRC would develop distant metastasis, and the 5-year survival rate is about 10% (Rao et al., 2018). KIRP comprises 15%–20% of renal cell cancer, which stems from the proximal nephron, the same origin with clear cell type (Malouf et al., 2016). Surgical treatment is also the main option for renal cancer. However, conventional therapeutic methods are the main treatment manners even though the results are not very satisfactory for those patients with metastases (Gill et al., 2017).

Iron is an essential element for the human body, and its metabolic disorder is involved in the occurrence and development of many diseases (Huang, 2003; Manz et al., 2016). Previous studies indicated that iron metabolism is no doubt the key role in the process of ferroptosis. Dixon et al. found that iron chelators could block ferroptotic cell death *in vitro* and *in vivo* (Dixon et al., 2012). They also found that exogenous iron administration could increase the induction of iron drop in cells (Dixon et al., 2012). Li et al. found that excessive heme and non-heme iron could directly induce ferroptosis (Yang et al., 2020; Zou et al., 2020). Ferroptosis is a newly coined non-apoptotic programmed cell death characterized by iron-dependent lipid peroxidation (Dixon et al., 2012; Chen et al., 2020). It is induced by the accumulation of ferric ions (Hirschhorn and Stockwell, 2019). Several signaling pathways or small molecules, such as MAPK, P53, Nrf2, erastin, and cisplatin, have been demonstrated to be involved in the regulation of ferroptosis (Dixon et al., 2012; Yu et al., 2015; Small et al., 2017; Xie et al., 2017; Guo et al., 2018). Dysregulated ferroptosis is associated with various diseases, including cancers, cardiovascular, neurodegenerative, and hepatic diseases (Han et al., 2020). The kidney is an organ related to iron metabolism, and several studies have shown that renal cancer is highly correlated with iron metabolism (Moon et al., 2019; Zhang et al., 2020).

Long noncoding RNAs (LncRNAs) are over 200 nucleotides in length, which accounts for nearly 70% of human transcriptome (Iyer et al., 2015). LncRNAs are increasingly recognized as the crucial mediators in the regulation of ferroptosis in various manners (Mou et al., 2019). For example, Wang et al. found that LINC00618 could inhibit ferroptosis by attenuating the expressions of lymphoid-specific helicase and solute carrier family 7 member 11 (Wang et al., 2021). Wang et al. found that overexpression of LINC00336 could inhibit ferroptosis by directly decreasing intracellular concentrations of iron and Fe^2+^, lipid reactive oxygen species (ROS), and mitochondrial superoxide (Wang M. et al., 2019). Ni et al. found that lncRNA-ZFAS1 could promote cardiomyocyte ferroptosis by downregulating Cyclin D2 through miR-150-5p (Ni et al., 2021). Accumulative studies have suggested that lncRNAs play pivotal roles in the progression of cancers and could be used as biomarkers to predict the outcome of cancers (Xiao et al., 2017; Zhai et al., 2017; Dong et al., 2019; You et al., 2019; Li et al., 2020). Therefore, the aim of our present study was to screen suitable ferroptosis-related lncRNAs as biomarkers to predict the outcome for renal cancer. The screened suitable biomarkers could be not only prognostic biomarkers but also potential therapeutic targets for renal cancer.

## Methods

### Data Acquisition and Processing

RNA-seq count data of renal cancer (KIRC (72 controls vs. 530 cancers) and KIRP (35 controls vs. 291 cancers)) and the corresponding clinical information were downloaded from an open database The Cancer Genome Atlas (TCGA) (https://portal.gdc.cancer.gov/). Renal cancer samples were randomly divided into the training group and validation group (Table 1) (Cai et al., 2021; Zhang et al., 2021). DESeq2 in R (3.6.2) was used to screen the differentially expressed genes (DEGs) with the specific criterion baseMean ≥ 100, |LogFoldChange| ≥ 1.0, and adj. *p* < 0.05. The lncRNAs and ferroptosis-related genes were downloaded from GENCODE (https://www.gencodegenes.org/) and FerrDb (http://www.zhounan.org/ferrdb), respectively. Spearman’s correlation analyses were used to screen the ferroptosis-related lncRNAs.

**TABLE 1:** Characteristics of renal cancer patients.

Characteristics	Entire cohort (*n* = 819)	Training group (*n* = 410)	Validation group (*n* = 409)
Age, years			
≤60	401	201	200
>60	418	209	209
Gender			
Female	262	129	133
Male	557	281	276
Stage			
Stage I	437	221	216
Stage II	78	38	40
Stage III	175	87	88
Stage IV	97	47	50
Unknown		17	15
T			
T1	464	237	227
T2	101	49	52
T3	239	117	122
T4	13	6	7
Unknown	2	1	1
N			
N0	289	147	142
N1	40	21	19
N2	4	2	2
Unknown	486	240	246
M			
M0	515	269	246
M1	87	41	46
Unknown	217	100	117
Vital			
Alive	602	304	298
Death	217	106	111

### Overall Survival Analyses and the Specific Model Construction

We utilized the survival packages in R (3.6.2) to carry out the Kaplan–Meier (K-M) survival analyses and univariate Cox regression analyses for ferroptosis-related differentially expressed lncRNAs (FR-DELs) to determine their overall survival (OS) relationship with renal cancer patients. Least absolute shrinkage and selection operator (LASSO) analyses were introduced to avoid overfitting. The FR-DELs filtered by the K-M analyses and univariate Cox regression analyses could be used as candidate biomarkers. Multivariate Cox regression analyses were performed for the candidate FR-DELs to eliminate the FR-DELs that may not be independent prognosis factors.

The specific prognosis model was constructed after multivariate Cox regression analyses, as follows (Xing et al., 2021): *Risk value* = *β*_*FR-DEL1*_ * *Exp*_*FR-DEL1*_ + *β*_*FR-DEL2*_ * *Exp*_*FR-DEL2*_ … + *β*_*FR-DELn*_ * *Exp*_*FR-DELn*_. The “Exp” means the expression of FR-DELs, and the “β” is the regression coefficient obtained from the multivariate Cox regression analyses.

The diagnostic model was constructed using the candidate signatures, as follows (Tang et al., 2020): Logit score = 0.675 + −0.044 * Exp_(DOCK8-AS1)_ + 0.095 * Exp_(SNHG17)_ + 0.120 * Exp_(RUSC1-AS1)_ + 0.145 * Exp_(LINC02609)_ + 0.109 * Exp_(LUCAT1)_.

### Protein Interaction Network and Functional Enrichment Analyses

We utilized the Search Tool for the Retrieval of Interacting Genes 11 (STRING 11) to assess the interactions with the default parameters (https://string-db.org/), and we utilized Cytoscape 3.7.2 to visualize the interactions. DAVID 6.8 was utilized to perform the Gene Ontology (GO) and Kyoto Encyclopedia of Genes and Genomes (KEGG) pathway enrichment analyses (https://david.ncifcrf.gov/).

### Principal Component Analyses and Statistical Analyses

We utilized the principal component analysis (PCA) to reduce the dimension and visualize the renal cancer patients with different risk values. A repeated-measures ANOVA followed by unpaired two-tailed Student’s t-test was used as indicated. All results are expressed as mean ± SEM.

## Results

### Identification of Ferroptosis-Related Differentially Expressed LncRNAs

There were 3,519 DEGs (Supplementary Figure S1A), including 2,382 upregulated DEGs and 1,137 downregulated DEGs, that had been screened by DESeq2. To determine which of these 3,519 DEGs are FR-DEGs, all of these 3,519 DEGs were overlapped with the recognized IR-DEGs. By overlapping analysis, we obtained 61 FR-DEGs (39 upregulated FR-DEGs and 22 downregulated FR-DEGs) (Supplementary Figure S1B). To determine which of these 3,519 DEGs are DELs, all of these 3,519 DEGs were overlapped with the recognized DELs. By overlapping analysis, we obtained 171 DELs (141 upregulated DELs and 35 downregulated DELs) (Supplementary Figure S1C).

To obtain FR-DELs, we introduced Spearman’s correlation analyses for those 61 FR-DEGs and 176 DELs. We found that 631 pairs of FR-DEGs-DELs had correlation R values greater than 0.5, involving 129 DELs and 42 FR-DEGs (Supplementary Table S1). We named those 129 DELs as 129 FR-DELs. We then performed protein interactions analyses for those 42 FR-DEGs, and the interactions are displayed in Supplementary Figure S2. The expressions of those 129 FR-DELs and 42 FR-DEGs are displayed in Supplementary Figure S3A and Supplementary Figure S3B, respectively.

### Development of Prognosis Signatures

**FIGURE 1:**
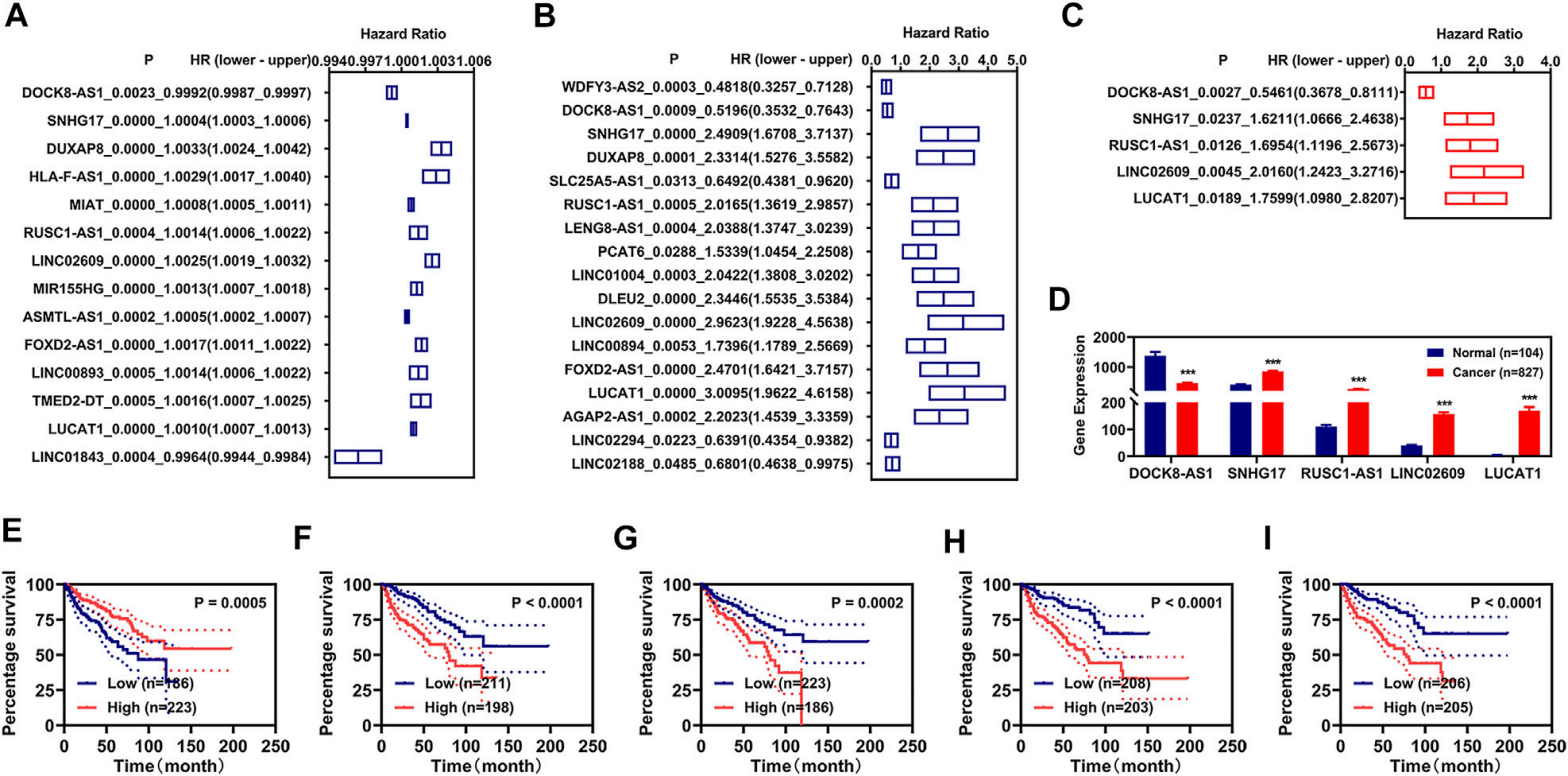
Identification of FR-DELs as prognosis signatures. **(A, B)**, Univariate Cox regression and K-M analyses illustrate that 14 FR-DELs **(A)** and 17 FR-DELs **(B)** were associated with OS in the training group, respectively. **(C)** Multivariate Cox regression illustrates that 5 FR-DELs were independently associated with OS in the training group. **(D)** The expression of those 5 FR-DELs in normal group and cancer group. **(E–I)** Kaplan–Meier curves of those 5 FR-DELs (DOCK8-AS1 **(E)**, SNHG17 **(F)**, RUSC1-AS1 **(G)**, LINC02609 **(H)**, and LUCAT1**(I)**) in renal cancers. *p < 0.05, **p < 0.01, ***p < 0.001. FR-DELs, ferroptosis-related differentially expressed lncRNAs; K-M, Kaplan–Meier; OS, overall survival.

To obtain suitable prognosis signatures for renal cancer in the training group, we firstly performed the univariate Cox regression analyses followed with LASSO analyses and identified 14 FR-DELs associated with the OS of renal cancer (Figure 1A, Supplementary Figures S4A,B). Similarly, we performed the K-M survival analyses followed by LASSO analyses and identified 17 FR-DELs associated with the OS of renal cancer (Figure 1B, Supplementary Figures S4C,D). By overlapping analyses, we obtained 7 FR-DEGs (DOCK8-AS1, SNHG17, DUXAP8, RUSC1-AS1, LINC02609, FOXD2-AS1, and LUCAT1) as candidate prognosis signatures. Then, we performed multivariate Cox regression analyses for those 7 FR-DELs and identified 5 FR-DELs (Figure 1C) independently associated with the OS of renal cancer. The expression of DOCK8-AS1 (Figure 1D) was significantly decreased, while the expressions of SNHG17, RUSC1-AS1, LINC02609, and LUCAT1 (Figure 1D) were increased significantly in patients with renal cancer. The patients with renal cancer with lower expression of DOCK8-AS1 displayed worse OS, and the patients with renal cancer with higher expressions of SNHG17, RUSC1-AS1, LINC02609, and LUCAT1 displayed worse OS (Figures 1E–I).

**FIGURE 2:**
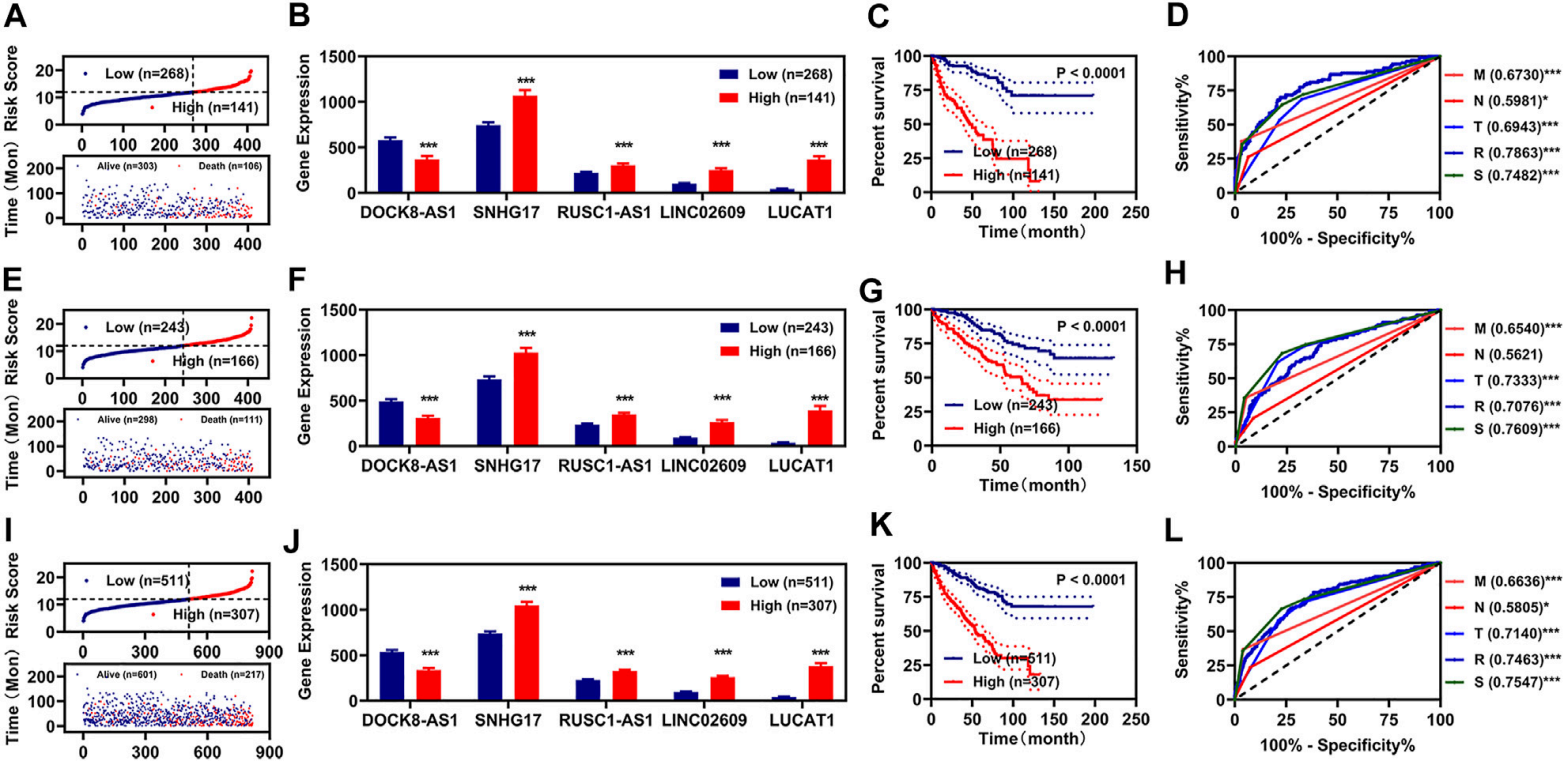
Development of prognosis signatures based on FR-DEMs and FR-DEGs. (A–D) Distribution of the risk values (up) and survival status (down) **(A)**, expression **(B)**, Kaplan–Meier curves **(C)**, and ROC curve **(D)** of the risk assessment model in the training group. **(E–H)** Distribution of the risk values (up) and survival status (down) **(E)**, expression **(F)**, Kaplan–Meier curves **(G)**, and ROC curve **(H)** of the risk assessment model in the validation group. **(I–L)** Distribution of the risk values (up) and survival status (down) **(I)**, expression **(J)**, Kaplan–Meier curves **(K)**, and ROC curve **(L)** of the risk assessment model in the entire group. *p < 0.05, **p < 0.01, ***p < 0.001. FR-DEMs, ferroptosis-related differentially expressed miRNAs; FR-DEGs, ferroptosis-related differentially expressed genes; ROC, receiver operating characteristic.

Subsequently, we utilized those 5 FR-DELs (DOCK8-AS1, SNHG17, RUSC1-AS1, LINC02609, and LUCAT1) to construct a risk assessment model in the training group. The risk values and survival status of each renal cancer patient are displayed in Figure 2A. We used Youden’s index as optima cutoff value to regroup the renal patients into the high-risk group and low-risk group (Supplementary Figure S5). The expressions of SNHG17, RUSC1-AS1, LINC02609, and LUCAT1 were significantly higher, while the expression of DOCK8-AS1 was lower in the high-risk group (Figure 2B). Patients with renal cancer with high-risk values displayed worse OS (Figure 2C). We also plot the receiver operating characteristic (ROC) curve and found that the area under the curve (AUC) of the risk model was comparable with that of the pathologic stage was slightly higher than that of the pathologic TNM (Figure 2D).

To determine whether those 5 FR-DELs (DOCK8-AS1, SNHG17, RUSC1-AS1, LINC02609, and LUCAT1) could be prognosis signatures for renal cancer, we used the validation group data and entire group data for verification. The risk model constructed by using those 5 FR-DELs (DOCK8-AS1, SNHG17, RUSC1-AS1, LINC02609, and LUCAT1) yielded similar results in the validation and entire group as in the training group (Figures 2E–L).

### Independent Prognosis Factors of Overall Survival

**FIGURE 3:**
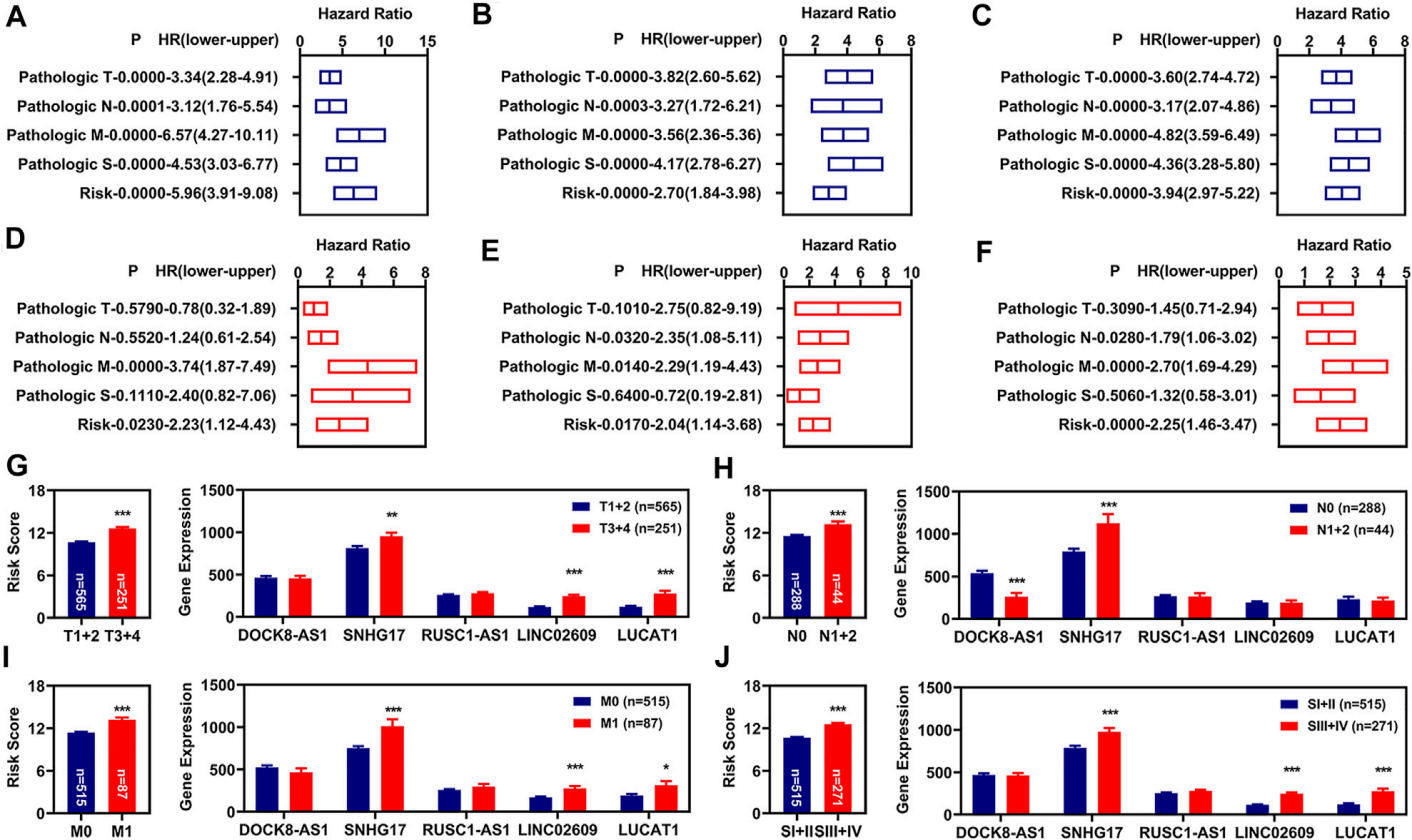
Independent prognosis factors and correlation analyses. **(A–C)** Univariate Cox regression of prognosis factors in training group **(A)**, validation group **(B)**, and entire group **(C)**. **(D–F)** Multivariate Cox regression of prognosis factors in training group **(D)**, validation group **(E)**, and entire group **(F)**. **(G–J)** Correlation of risk values (left) and those 5 FR-DELs (right) with the pathologic T **(G)**, pathologic N **(H)**, pathologic N **(I)**, and pathologic stage **(J)**. *p < 0.05, **p < 0.01, ***p < 0.001. FR-DELs, ferroptosis-related differentially expressed lncRNAs.

To determine whether this risk model could be independently used for prognosis diagnosis, K-M and multivariate Cox regression analyses for clinicopathological features and the risk model were performed. In the training group, we found that the pathologic TNM, pathologic stage, and risk model were significantly correlated with the OS of renal cancer as measured by K-M analyses (Figure 3A). The pathologic M and the risk model were still correlated with the OS of renal cancers as measured by multivariate Cox regression analyses (Figure 3D). To determine whether our risk assessment was feasible, we used validation group data and entire group data for verification. In the validation group and entire group, we also found that the pathologic TNM, pathologic stage, and risk model were also significantly correlated with the OS of renal cancer (Figures 3B,C). Meanwhile, the pathologic NM and the risk model were independently correlated with the OS of renal cancers in the multivariate Cox regression analyses (Figures 3E,F).

By retrospective examination of the ROC curves in the entire group, we found that although both the pathologic NM and the risk model were independently associated with OS of renal cancer, the AUC values of the pathologic NM were lower than those of the risk model (Figure 2L). We then performed ROC curves analyses in the entire group at 1, 3, 5, and 10 years. All AUC values were over 0.7 (Supplementary Figure S6).

Subsequently, we analyzed the risk values in different clinicopathological feature groups. There were significant differences for risk values in different pathologic TNM and pathologic stage (Figures 3G–J, left). We also analyzed the expressions of those 5 FR-DELs (DOCK8-AS1, SNHG17, RUSC1-AS1, LINC02609, and LUCAT1) in different clinicopathological feature groups, and the results are displayed in Figures 3G–J, right.

### Principal Component Analysis and Enrichment Analyses

**FIGURE 4:**
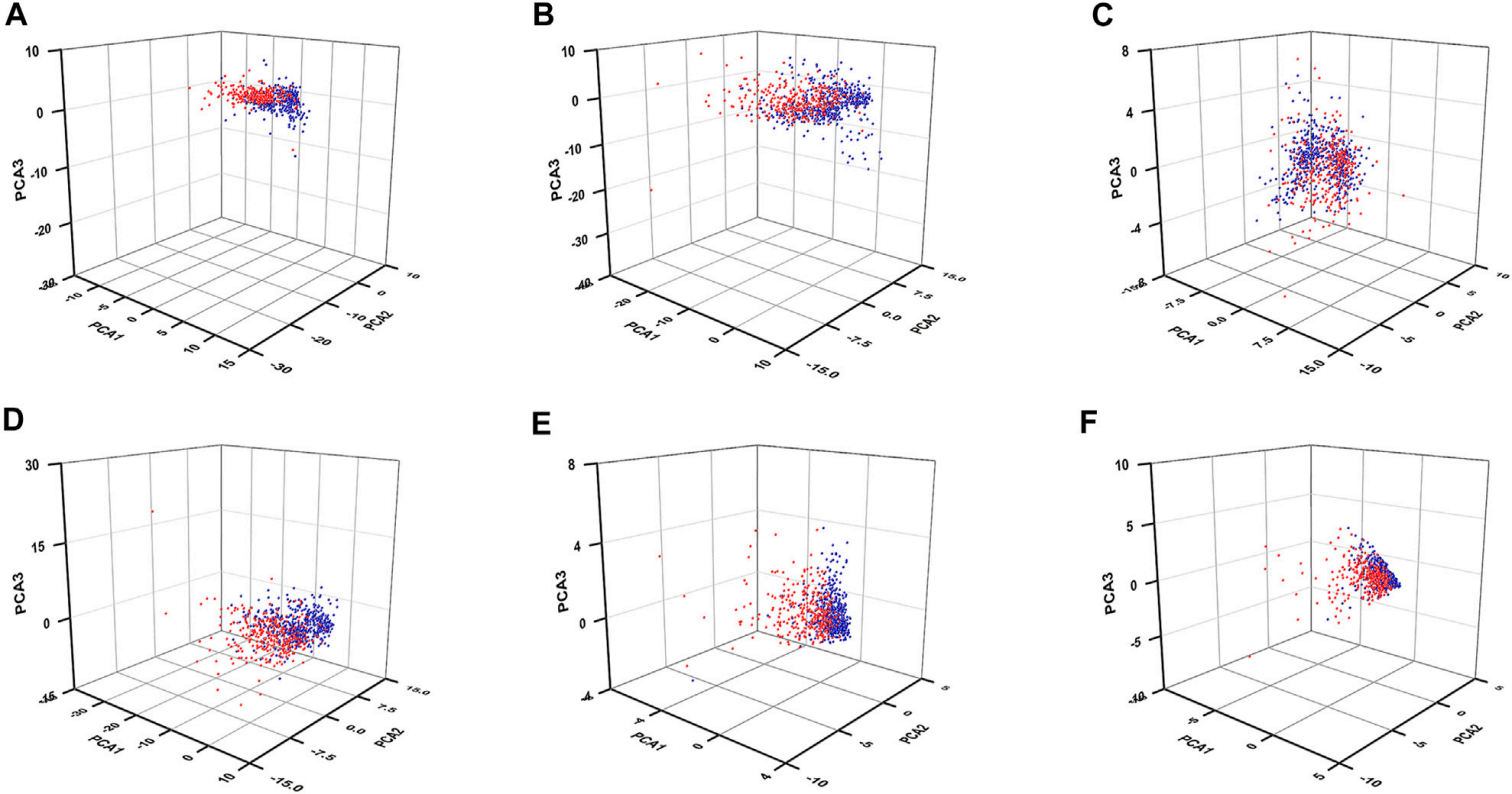
PCA for renal cancer patients with different risk values. PCA plots display the distribution of patients with renal cancer with high-risk value and low-risk value based on 61 FR-DEGs **(A)**, 176 DELs **(B)**, 42 FR-DEGs **(C)**, 129 FR-DELs **(D)**, 7 FR-DELs **(E)**, and 5 FR-DELs **(F)**. Blue means low risk. Read means high risk. PCA, principal component analysis; FR-DEGs, ferroptosis-related differentially expressed genes; DELs, differentially expressed lncRNAs; FR-DELs, ferroptosis-related differentially expressed lncRNAs.

To know whether those DEGs could be used to distinguish the high-risk patients from the low-risk patients, we performed PCAs by using those 61 FR-DEGs (filtered by DEGs crossed with ferroptosis-related genes), 176 FR-DELs (filtered by DEGs crossed with lncRNAs), 42 FR-DEGs (filtered by Spearman’s correlation analyses), 129 FR-DELs (filtered by Spearman’s correlation analyses), 7 FR-DELs (filtered by univariate Cox regression analyses and K-M survival analyses), and 5 FR-DELs (filtered by multivariate Cox regression analyses). As we can see from Figures 4A–E, the renal cancer patients with high-risk values are basically distinguishable from those with low-risk patients by using those DELs. Particularly, we found that the distribution of renal cancer patients with low-risk values was relatively concentrated, which could be well distinguished from renal cancer patients with high-risk values by using those 5 FR-DELs (DOCK8-AS1, SNHG17, RUSC1-AS1, LINC02609, and LUCAT1) (Figure 4F).

We then performed differential expression analyses between high-risk renal cancer patients and low-risk renal cancer patients and found that 460 DEGs were upregulated and 381 DEGs were downregulated (Supplementary Figure S7). We performed the protein interaction and functional enrichment analyses for those 841 DEGs. If a gene is connected with more genes, it is considered generally to have a more important role. In the present study, we obtained 206 central DEGs by using the average node degree (9.25) as the criterion. The interaction of those 206 DEGs is displayed in Supplementary Figure S8.

**FIGURE 5:**
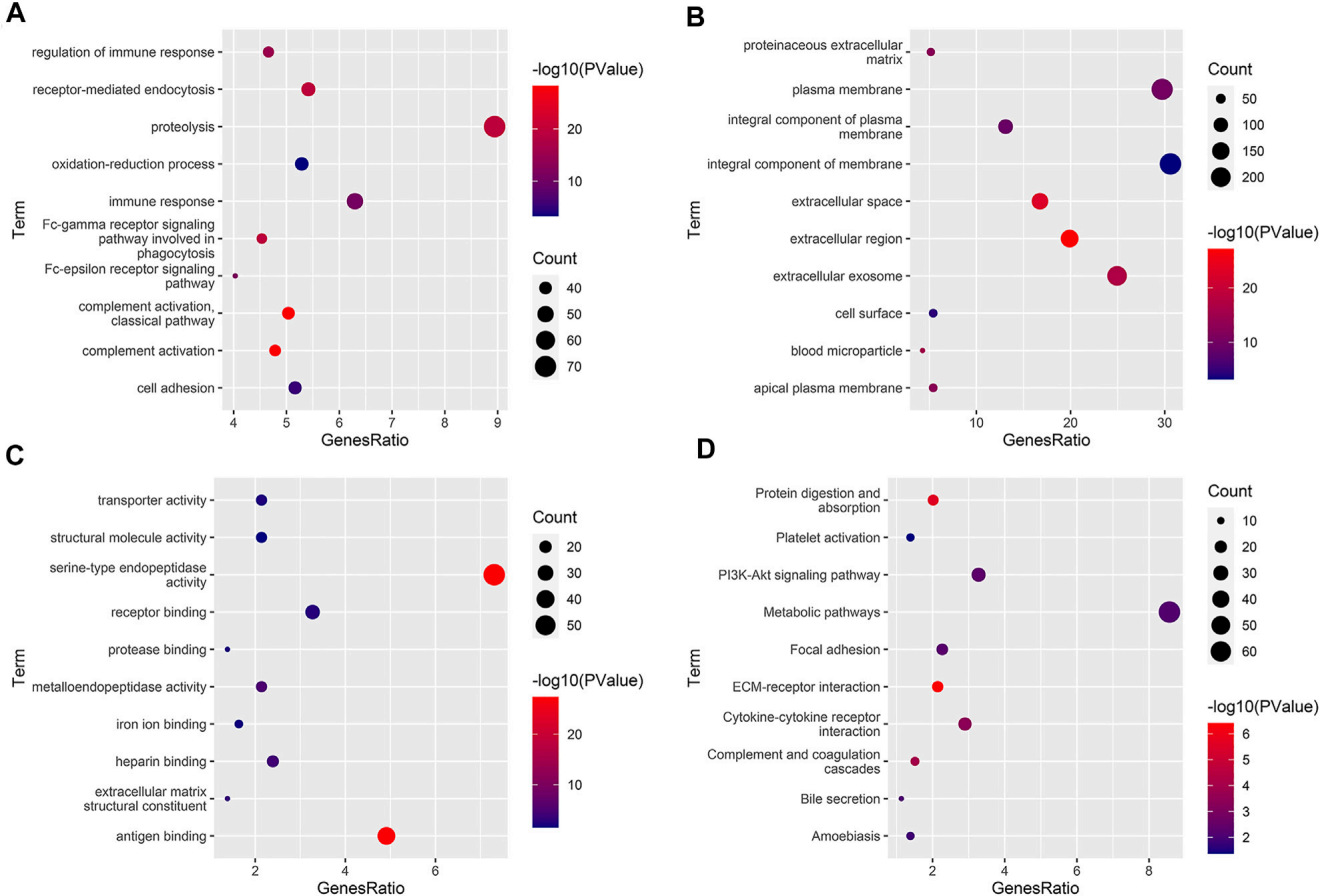
Functional enrichment analyses. **(A–C)** The significantly enriched GO term (top 10). BP, biological process **(B)**. CC, cellular component **(B)**. MF, molecular function **(C)**. **(D)** The significantly enriched KEGG pathway (top 10). GO, Gene Ontology; KEGG, Kyoto Encyclopedia of Genes and Genomes.

**FIGURE 6:**
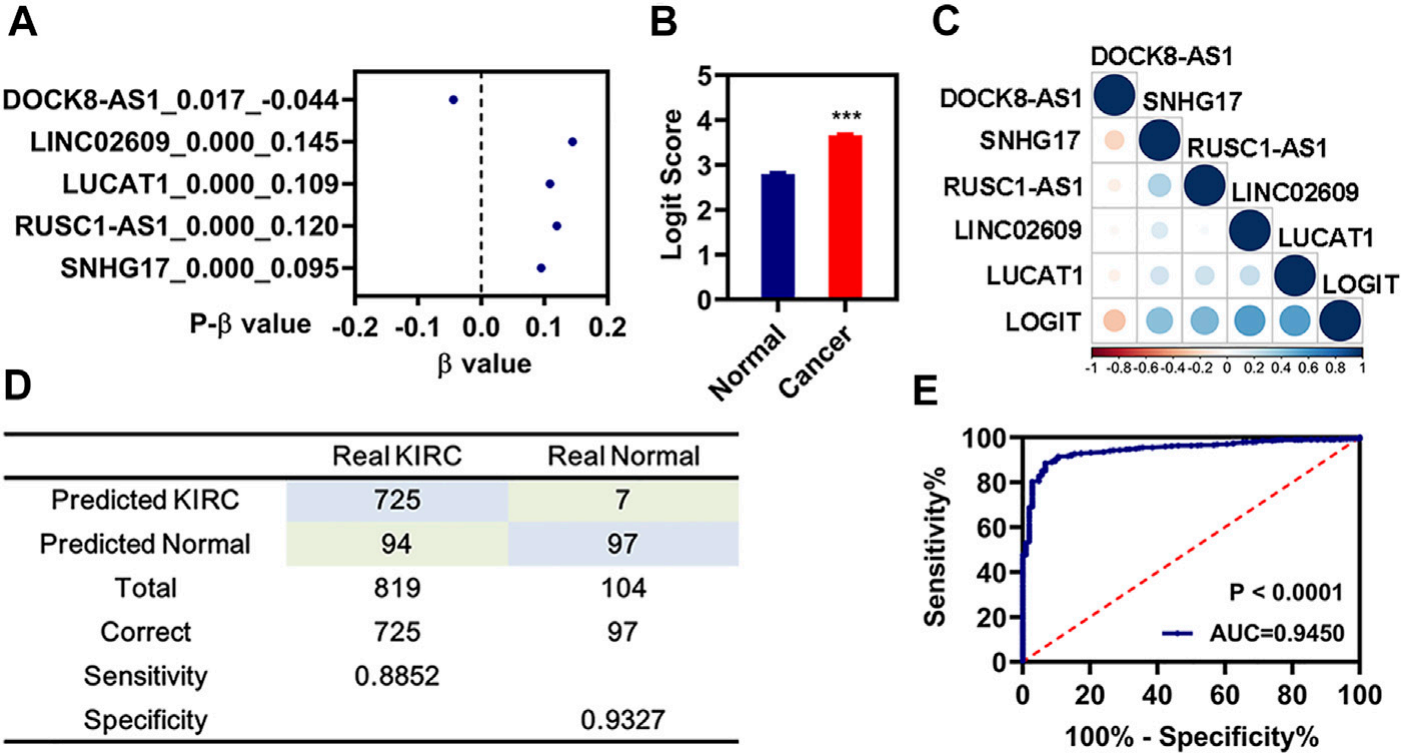
Diagnosis model constructed by those 5 FR-DELs. **(A)** β value of those 5 FR-DELs analyzed by stepwise logistic regression. **(B)** Diagnostic values between cancer and normal. **(C)** Correlation analysis for the expression of those 5 FR-DELs and diagnostic values. **(D)** Construction matrix of the classification results of the diagnostic model. **(E)** ROC curves for evaluating the predictive performance of the diagnostic model. *p < 0.05, **p < 0.01, ***p < 0.001. FR-DELs, ferroptosis-related differentially expressed lncRNAs; ROC, receiver operating characteristic.

GO analyses indicated that 171 biological processes (BPs), 39 cellular components (CCs), and 65 molecular functions (MFs) were enriched, of which 120 BPs, 29 CCs, and 45 MFs were enriched significantly (Supplementary Table S2). The significantly enriched BPs, CCs, and MFs with the number of genes ranked in the top 10 are shown in Figure 5A and Figure 6C. KEGG analyses indicated that 27 signaling pathways were enriched, of which 18 signaling pathways were enriched significantly (Supplementary Table S3). The significantly enriched signaling pathways with the number of genes ranked in the top 10 are shown in Figure 5D.

### Construction of the Diagnostic Model

A diagnostic model integrating those 5 FR-DELs (DOCK8-AS1, SNHG17, RUSC1-AS1, LINC02609, and LUCAT1) was established to separate patients with renal cancer from the normal group by using a stepwise logistic regression method. Diagnostic scores were identified as follows: Logit score = 0.675 + −0.044 * Exp_(DOCK8-AS1)_ + 0.095 * Exp_(SNHG17)_ + 0.120 * Exp_(RUSC1-AS1)_ + 0.145 * Exp_(LINC02609)_ + 0.109 * Exp_(LUCAT1)_ (Figure 6A)_._ The logit value of patients with renal cancer was significantly higher than that of the normal by t-test analysis (Figure 6B). The expression of DOCK8-AS1 was significantly decreased, while the expressions of those 4 signatures (SNHG17, RUSC1-AS1, LINC02609, and LUCAT1) were significantly increased in the patients with renal cancer (Supplementary Table S4). The correlation analysis indicated that the expressions of those 4 FR-DELs (SNHG17, RUSC1-AS1, LINC02609, and LUCAT1) were significantly correlated with the logit value (Figure 6C). The sensitivity and specificity of the diagnostic model were 88.52% and 93.27%, respectively (Figure 6D). We also plot the ROC curve of the diagnostic model, and the AUC value was 0.9450.

## Discussions

Renal cancer is the second most common cancer of the urinary system after bladder cancer. About 40% of patients with advanced renal cancer would develop distant metastasis and have a poor prognosis, with the 5-year prognosis being about 10% (Rao et al., 2018). For those patients with metastatic tumors, conventional therapeutic methods are the main treatments. However, these results are not very satisfactory (Gill et al., 2017). The developments of new therapeutic methods and suitable prognosis signatures have important practical clinical significance.

Ferroptosis is a novelty recognized process of regulated cell death, and accumulative evidences indicate that it could be used as a new therapeutic target for cancers (Huang et al., 2005; Liu et al., 2011; Roh et al., 2016). Additionally, previous studies also indicated that lncRNAs are involved in the development of cancers and could regulate ferroptosis (Xiao et al., 2017; Zhai et al., 2017; Dong et al., 2019; Mou et al., 2019). To obtain suitable signatures closely related to survival, we integrated univariate Cox regression analyses, K-M analyses, and LASSO analyses. In order to increase the clinical availability of these candidate signatures, we constructed and assessed the diagnostic model and prognostic model using them. Finally, we identified 5 FR-DELs (DOCK8-AS1, SNHG17, RUSC1-AS1, LINC02609, and LUCAT1) correlated with the OS of renal cancer patients independently through a series of bioinformatics analyses. The ROC curve suggested that the risk assessment model constructed by those 5 FR-DELs (DOCK8-AS1, SNHG17, RUSC1-AS1, LINC02609, and LUCAT1) could be used for prognosis signatures for renal cancer patients.

In the present study, we found that the expressions of SNHG17, RUSC1-AS1, LINC02609, and LUCAT1 were significantly increased while the expression of DOCK8-AS1 was significantly decreased in the patients with renal cancer and in renal cancer patients with high-risk values. Renal cancer patients with low DOCK8-AS expression displayed worse OS. At present, there are no studies on DOCK8-AS and cancers. Therefore, further researches are needed to find out whether DOCK8-AS could be related to renal cancer and be used for the prediction of renal cancer. Previous studies have demonstrated that the expressions of SNHG17, RUSC1-AS1, LINC02609, and LUCAT1 were significantly increased in many cancers. For example, SNHG17 has been found to be significantly higher in breast cancer, colorectal cancer, gastric cancer, and prostate cancer (Zhang et al., 2019; Du et al., 2020; Liu et al., 2020a; Zhao et al., 2021). It could regulate the progression of cancer in various manners. Accumulative studies have demonstrated that SNHG17 could promote the proliferation, migration, and invasion by inhibiting miRNAs (miR-23a-3p, miR-124-3p, and miRNA-375-3p) (Du et al., 2020; Liu et al., 2020a; Cao et al., 2021), by epigenetically silencing p15 and p57 (Zhang et al., 2019) and by β-catenin signaling pathway (Zhao et al., 2021). In the present study, we found that SNHG17 was correlated with the pathologic TNM and pathologic stage and correlated with the OS of renal cancer, which was consistent with previous reports (Zhang et al., 2019; Zhao et al., 2021). For example, the high expression of SNHG17 was associated with the increased invasion depth, lymph node metastasis, and advanced TNM stage of gastric cancer (Zhang et al., 2019). SNHG17 high expression was associated with poor outcomes in patients with prostate cancer (Zhao et al., 2021). RUSC1-AS1 has been found to be significantly higher in cervical cancer, breast cancer, and osteosarcoma (Hu et al., 2019). Guo et al. found that the expression of RUSC1-AS1 was significantly increased in the cervical cancer tissues and cell lines (Guo et al., 2020). High expression of RUSC1-AS1 could promote the proliferation, apoptosis, migration, and invasiveness of cervical cancer cells (Guo et al., 2020). Hu et al. also found that the expression of RUSC1-AS1 was significantly increased in the breast cancer tissues, and the silence of RUSC1-AS1 could inhibit viability, clonality, cell cycle progression, and apoptosis of breast cancer cells (Hu et al., 2019). Additionally, a previous study also demonstrated that the expression of RUSC1-AS1 was correlated with OS. Patients with cervical cancer with high expression of RUSC1-AS1 displayed worse OS (Guo et al., 2020). Our study also found that RUSC1-AS1 was significantly correlated with renal cancer, which reinforced the pivotal role of RUSC1-AS1 in cancers. Su et al. found that the expression of LINC02609 was not only significantly higher in advanced stages and grades than in early stages and grades but also significantly higher in tumor and distant metastatic tissues than in normal and non-distant metastatic controls (Su et al., 2021). In the present study, we also found that the expression of LINC02609 was significantly different in differential clinicopathological features. It was significant in the patients with renal cancer with pathologic T3+4, pathologic M1, and pathologic stage III+IV. Our present study also indicated that LINC02609 was correlated with metastatic. Accumulative evidences have shown that LUCAT1 is involved in the progression of several cancers. It is highly expressed in liver cancer, colorectal cancer, and ovarian cancer. LUCAT1 could promote proliferation, migration, and invasion of cancer cells in various manners, such as inhibiting KISS1 expression (Liu et al., 2019), targeting KLF6 and KLF15 (Liu et al., 2020b), and targeting the L40-MDM2-p53 pathway through binding with UBA52 (Zhou et al., 2019). For the treatment of colorectal, Lin et al. found that chemotherapeutic agents combined with antisense oligonucleotides downregulated LUCAT1 yielded better results than the chemotherapeutic agents alone (Huan et al., 2020). Therefore, it is necessary to determine the expression of LUCAT1 for the treatment of colorectal cancer. Moreover, previous studies indicated that LUCAT1 could be a potential prognostic biomarker for patients with several cancers (Yoon et al., 2018; Yu et al., 2018; Zhou et al., 2019; Huan et al., 2020; Yang and Xia, 2020), including renal cancer (Wang et al., 2018; Zheng et al., 2018; Wang Y et al., 2019). In the present study, we also found that LUCAT1 could use as a prognostic biomarker for renal cancer.

## Conclusion

In the present study, we identified 5 FR-DELs (DOCK8-AS1, SNHG17, RUSC1-AS1, LINC02609, and LUCAT1) that could be used as biomarkers to predict the outcome of renal cancer. We will carry out the functional and clinical verification research for those 5 FR-DELs to see the feasibility of clinical applications in the next steps. Our study may also provide a new research strategy for exploring the ferroptosis-related signatures for cancers.

## Data Availability

Publicly available datasets were analyzed in this study. These data can be found here: https://portal.gdc.cancer.gov/.
